# The Role of Extracellular Adenosine in Chemical Neurotransmission in the Hippocampus and Basal Ganglia: Pharmacological and Clinical Aspects

**DOI:** 10.2174/156802611795347564

**Published:** 2011-04

**Authors:** Beáta Sperlágh, E. Sylvester Vizi

**Affiliations:** Department of Pharmacology, Institute of Experimental Medicine, Budapest, Hungary and Institute of Pharmacology and Pharmacotherapy, Semmelweis University, Budapest, Hungary

**Keywords:** Adenosine, A_1_ receptor, A_2A_ receptor, ATP, hippocampus, striatum.

## Abstract

Now there is general agreement that the purine nucleoside adenosine is an important neuromodulator in the central nervous system, playing a crucial role in neuronal excitability and synaptic/non-synaptic transmission in the hippocampus and basal ganglia. Adenosine is derived from the breakdown of extra- or intracellular ATP and is released upon a variety of physiological and pathological stimuli from neuronal and non-neuronal sources, i.e. from glial cells and exerts effects diffusing far away from release sites. The resultant elevation of adenosine levels in the extracellular space reaches micromolar level, and leads to the activation A_1_, A_2A_, A_2B_ and A_3_ receptors, localized to pre- and postsynaptic as well as extrasynaptic sites. Activation of presynaptic A_1_ receptors inhibits the release of the majority of transmitters including glutamate, acetylcholine, noradrenaline, 5-HT and dopamine, whilst the stimulation of A_2A_ receptors facilitates the release of glutamate and acetylcholine and inhibits the release of GABA. These actions underlie modulation of neuronal excitability, synaptic plasticity and coordination of neural networks and provide intriguing target sites for pharmacological intervention in ischemia and Parkinson’s disease. However, despite that adenosine is also released during ischemia, A_1_ adenosine receptors do not participate in the modulation of excitotoxic glutamate release, which is nonsynaptic and is due to the reverse operation of transporters. Instead, extrasynaptic A1 receptors might be responsible for the neuroprotection afforded by A_1_ receptor activation.

## INTRODUCTION

Since the first suggestion by Drury and Szent-Györgyi in 1929 [1] that extracellular adenosine may have a physiological effect on living cells, forty-one years elapsed before it was shown that adenosine acts at the subcellular level to increase cAMP [2]. Studies have also shown that adenosine is released [3] from cortical slices and inhibits acetylcholine (ACh) release from cholinergic terminals evoked by axonal stimulation via activation of theophylline-sensitive receptors [4, 5]. The finding [5] that theophylline competitively inhibited the presynaptic inhibitory effect of adenosine (Ki = 21 μM) and nucleotides has triggered studies using purinergic transmission and changed the dogma that nucleotides might have exclusive effects on postsynaptic sites.

In 1978, Burnstock proposed [6] that there were two purinergic receptors: adenosine-sensitive P_1_ and ATP/ADP-sensitive P_2_ receptors. Burnstock concluded that P_1_ receptors were located presynaptically and P_2_ receptors were located on the postsynaptic site. A couple of years later, studies found that both ATP and its non-metabolising compound α,β-methylene ATP were able to act presynaptically on P_2X_ receptors on the nerve terminals to potentiate transmitter release [7-9].

The general view is that adenosine is not a transmitter substance. It is not synthesised and/or stored in vesicles and is not released from nerve terminals in response to depolarisation followed by Ca^2+^-influx, although recent studies raised the possibility that adenosine could be also released in a vesicular fashion [10]. It is the degradation product of ATP in the extracellular space and may act as a chemical messenger at ambient concentrations. Therefore, it is generally accepted that the presence of ATP and its metabolite adenosine in the extracellular space may function in the central nervous system as nonsynaptic signalling molecules that diffuse far away from the cells where they originated from and tonically influence chemical neurotransmission [11], inflammation [12, 13] and immune responses [14]. Although the role of ATP in immunity is closely related to the role of its breakdown products, i.e., the nucleoside adenosine, as far as the chemical neurotransmission is concerned, adenosine and nucleotides such as ATP, ADP and AMP all have their own presynaptic effects on chemical neurotransmission [5, 9]. These molecules can inhibit or facilitate the release of transmitters via activation of P_1_ or P_2X_ receptors. To date, two families of purinergic receptors have been defined, P_1_ and P_2_ receptors [15]. Adenosine acts on P_1_ receptors, which are subdivided into G-protein-coupled A_1_, A_2A_, A_2B_ and A_3_ receptor subtypes [16]. The primary second messenger of all four subtypes of adenosine receptors is adenylate cyclase, which is either activated or inhibited depending on the type of receptor that is stimulated. A_1_ and A_3 _receptors are coupled to G_i_, and their activation results in a decrease of cAMP levels, increase of K^+^ conductance, and decrease of transient Ca^2+^ conductance important in transmitter release. These effects are analogous to the effect of presynaptic M_2_ acetylcholine receptors or α _2_ adrenergic receptors, both of which are G_i_-coupled receptors. Adenosine A_2A_ and A_2B_ receptor subtypes, however, are coupled to G_s_ and increase levels of cAMP.

## SOURCE OF EXTRACELLULAR ADENOSINE

The role of extracellular space has been emphasized in nonsynaptic chemical interactions [11]. Extracellular adenosine is thought to be generated in the extracellular space either as a breakdown product of released ATP [17] or as the released adenosine, depending on the type of stimulus (e.g. during ischemia, [18]). Because ATP is ubiquitous, all metabolically active cells of the nervous system are able to synthesise ATP, which provides a potential pool for the release of adenosine. Therefore, the cellular source of released purines in the hippocampus and basal ganglia could be any cell type present in these brain areas (i.e., nerve terminals, astrocytes and other types of glia, microglial cells and endothelia). The majority of ATP is formed in the mitochondria by oxidative phosphorylation, which results in approximately 5-10 mM ATP concentration in the cytoplasm under normal metabolic conditions. In addition, ATP has also been shown to be taken up and stored in synaptic vesicles of nerve terminals [19] and astrocytes [20]. There is, however, intense controversy about whether astrocytes can exocytose transmitters *in vivo* [21]. Conversely, the basal extracellular adenosine concentration was much lower (in the low-micromolar to high-nanomolar range), and the majority of adenosine was taken up into cells and rapidly reincorporated into ATP stores or deaminated by adenosine deaminase under normal metabolic conditions [21, 22].

A wide variety of stimuli are known to release ATP and/or adenosine to the extracellular space, which could theoretically lead to sufficient purine levels to activate either ATP or adenosine receptors [19, 22, 23]. Indeed, ATP and adenosine have been shown to be released in response to KCl depolarisation [24] low- [25, 26] and high-frequency [25, 27, 28] electrical stimulation from acute hippocampal and striatal slices and in response to glutamate receptor activation [29]. Interleukin-1β (IL-1β), a cellular mediator of inflammation is able to release ATP from hippocampal slices [30]. Generally, it appears that the proportion of adenosine derived from the extracellular breakdown of ATP is higher when the stimulation frequency is higher [25]. ATP and various transmitters are released from nerve terminals when exocytosis occurs in response to neuronal firing because neurotransmitters are stored together with ATP in the vesicles. ATP could also be released from postsynaptic sites in response to activation of receptors by primary transmitters [31]. Various purines, including ATP and adenosine, are also released from the hippocampus and striatum *in vivo* by the quasi-physiological stimuli mentioned above [32-35]. It is also important to mention that depolarising stimuli lead to the extracellular accumulation of not only adenine nucleotides and nucleosides but also guanine and pyrimidine nucleosides, such as uridine, which might also act as neuroactive substances under certain circumstances [36-38]. 

Although the stimulation-dependent release of ATP and adenosine by conventional neuronal activity is well documented, these stimuli probably result in a spatially restricted, localised increase in extracellular purine levels, which contribute to the synaptic transmission and the modulation of pre- and post-synaptic functions within the synaptic cleft. Thus, ATP-metabolising ectoenzymes, present on the nerve terminal membrane, and nucleoside transporters may strongly limit further purine availability.

Pathological events are also known to powerfully stimulate purine release. These signals include mechanical [20, 39, 40] and hypotonic [41] stimuli, hypoxia/ hypoglycemia/ ischemia and consequent energy deprivation [18, 26, 42-44], inflammatory signals, such as bacterial lipopolysaccharide (LPS) [45, 46], and cytolysis. Among pathological stimuli, hypoxia and ischemic-like conditions are well-known causes of adenosine release in the striatum and hippocampus both *in vitro* [18, 47-53] and *in vivo* [54-58] (for further reference see [22]). However, unlike extracellular adenosine accumulation detected in response to physiological stimuli, the source of adenosine released by metabolic distress is mainly intracellular [59]. Pathological signals could release purines from neurons and non-neuronal cells. This release would result in a purine-rich extracellular milieu that could lead to a widespread activation of receptors that may reach neighbouring or distant cells, such as astrocytes and microglia. Moreover, nucleotides and nucleosides themselves may promote further release of purines, by a homo- or heteroexchange mechanism, if they reached relatively high concentrations in the extracellular space [60].

If purines are in the extracellular space, then their extrasynaptic concentrations are controlled by the enzymes that catalyse their conversion [61]. Several enzyme families are responsible for the extracellular degradation of ATP in the nervous system. The first step of the inactivation of ATP is mediated by the family of ectonucleoside triphosphate diphosphohydrolases (E-NTPDases, EC 3.6.1.5, also known as ectoATPase or apyrase), which are able to hydrolyse ATP and ADP to AMP [61]. These enzymes show widespread distribution in the brain [62, 63] and have micromolar K_m_ for ATP and ADP, which causes rapid and highly effective hydrolysis of ATP in almost all neuronal tissues (Table **1**). In addition to the E-NTPDase family, ATP can also be dephosphorylated by ecto-nucleotide pyrophosphatases (E-NPPs) and alkaline phosphatases, which both have broader substrate specificity and widespread tissue distribution [61]. The next step of extracellular inactivation is the hydrolysis of AMP by the ecto-5`nucleotidase (EC 3.1.3.5) (CD73) enzyme, which is the rate-limiting step in the formation of adenosine [19, 64]. Ecto-5’-nucleotidase exhibits a micromolar K_m_ for AMP and is feed-forwardly inhibited by ATP, which results in a delayed, ‘burst-like’ adenosine production [65]. It is also widely expressed in the brain, and it is predominantly associated to glial cells [66, 67]; however, its expression has also been demonstrated in purified hippocampal and striatal nerve terminals [68, 69]. Adenosine deaminase and adenosine kinase are the key enzymes in adenosine metabolism. Adenosine deaminase catalyses the deamination of adenosine to inosine [70], and adenosine kinase catalyses the phosphorylation of adenosine to AMP. Microglial cells are brain-specific macrophages that are able to release adenosine, produce it through ATP breakdown and respond to extracellular adenosine Fig. (**1**). Microglial cells are also equipped with different adenosine receptors [71]. They express functional A_1_, A_2A_ and A_3_ but not A_2B_ adenosine receptors [71], which regulate different aspects of the microglial response [13].

Adenosine-mediated actions can be terminated by its uptake into the cells via specific nucleoside transporters, which are widely expressed in the nervous system. Specific nucleoside transporters consist of two families: equilibrative transporters (ENTs) and concentrative transporters (CNTs). ENTs are driven by the concentration gradient, and CNTs are driven by the sodium gradient [72, 73]. ENTs can carry different nucleosides, including adenosine and inosine, but not nucleotides, across the cell membrane in both directions. They are regarded as the dominant nucleoside transporters of the brain. Because the intracellular adenosine level under normal metabolic conditions is in the low micromolar range [22], ENTs loaded from the extracellular space by excess adenosine mediate adenosine uptake into the nerve terminals. When adenosine is taken up, the adenosine kinase and adenosine deaminase enzymes convert it to AMP and inosine, respectively, thereby maintaining the driving force of the carrier. However, the ENTs can also act in a reverse direction under certain circumstances, which mediates the release of adenosine into the extracellular space. Excessive accumulation of adenosine could occur during energy deprivation or metabolic distress when ATP stores are depleted and AMP is generated intracellularly. Cytosolic 5’nucleotidase, which has a relatively high K_m_ for AMP (1-14 μM), becomes active under energy deprivation and accumulates adenosine intracellularly. This adenosine then flows out to the extracellular space in a transporter mediated manner [22, 65].

## EXTRACELLULAR CONCENTRATIONS OF ADENOSINE

The availability of extracellular adenosine is determined by a balance between the speed of decomposition of ATP by ectonucleotidases [64] and the rate of release of ATP and adenosine. Extracellular ATP is decomposed to adenosine in the hippocampus with a half-life of about 200 msec [17]. The maintenance of an ambient concentration of adenosine in the extracellular space is mainly dependent on ATP release from astrocytes [74]. Once ATP is released into the extracellular space, it can be rapidly metabolised (within seconds, [17]) to adenosine, which is normally present in a concentration between 10 and 30 nM. Under hypoxia or ischemia, adenosine concentrations can reach 20 [17] and 30 μM [75], respectively, in the extracellular space of the hippocampus. According to Zetterström *et al.* (1982) [58], adenosine concentrations under resting conditions can be as high as 1-2 μM. One *in vivo *study observed adenosine levels around 19- to 23-fold compared with resting levels in response to ischemia [55]. Extracellular concentrations of adenosine and ATP of neuronal or glial origin can increase markedly in response to inflammation [22]. Once released into the extracellular space, adenosine may diffuse far away and reach nonsynaptic A_1_ receptors as well as high-affinity nonsynaptic A_2A_ receptors. Therefore, the balance between A_1_-receptor-mediated neuroprotective and A_2A_ receptor-mediated excitotoxic effects plays an important role in the outcome of adenosine action [76]. At lower concentrations of adenosine, the A_1_ receptors were mainly tonically activated and A_2A_ receptors were not stimulated [77]. However, neuronal activity increased the bursting rate and the concentration of adenosine in the extracellular space, which caused both receptors to get tonically activated. It was observed that the stimulation of A_2A_ receptors by adenosine, formed from ATP at high-frequency neuronal firing, caused a down-regulation of the A_1_ receptors. This finding supports observations that A_2A_ receptor blockade potentiates the neuroprotective actions of A_1_ receptor activation [78]. 

## EFFECT OF ADENOSINE ON TRANSMITTER RELEASE

It is generally accepted that adenosine plays a crucial role in neuronal excitability and synaptic transmission in the CNS. Electrophysiological and neurochemical evidence showed that presynaptic A_1_ receptor activation reduced the release of different transmitters in the CNS including the hippocampus and basal ganglia (Table **2**). The adenosine A_1_ receptors are widely distributed in the CNS, and their activation induces several responses. These responses include inhibition of voltage dependent Ca^2+^ channels, activation of K^+^ channels resulting in hyperpolarisation and inhibition of adenylyl cyclase, all of which may cause inhibition of transmitter release. Furthermore, evidence was observed that nerve terminals were equipped with A_2A_ receptors, and stimulation of these receptors increased the release of different transmitters.

### Electrophysiological Evidence

Activation of A_1_ receptors decreases synaptic transmission in the hippocampus [79]. The stimulation of A_1_ receptors located on Schaffer collateral axon terminals by adenosine decomposed from ATP inhibited the field EPSPs and EPSCs in hippocampal slices [80]. This finding indicated that the release of glutamate (Glu) Fig. (**1**) was inhibited from the hippocampus [81, 82]. Other studies also shed light on that endogenous adenosine, involved in the tonic A_1_ receptor mediated inhibition of glutamatergic transmission, has largely astrocytic origin. Astrocytes are able to release ATP in a vesicular fashion [20], which then broke down to adenosine by the ectonucleotidase cascade [74]. Activation of hippocampal astrocytic network leads to heterosynaptic depression of excitatory transmission, a mechanism, whereby the stimulation of a particular pathway could suppress the activation of a nearby pathway and coordinate the activity of a given network [83]. Heterosynaptic depression is mediated by A_1_ adenosine receptors and selective gliotoxins [84] or selective knockdown of exocytotic machinery in astrocytes [74] leads to the disappearance of A_1_-receptor mediated inhibition of excitatory transmission and the heterosynaptic depression in the hippocampal slices.

In this preparation, A_1_ and CB1 cannabinoid receptors were colocalised on glutamatergic terminals [80]. A_1_ receptor activation by endogenous adenosine (e.g., increased in concentration during ischemia or hypoxia) prevented the CB1 receptor-mediated reduction of Glu release. Accordingly, A_1_ receptor antagonism with caffeine potentiated the effects of endocannabinoids [80], which indicated that cannabinoid signalling and presynaptic control of glutamatergic transmission in the hippocampus was limited by the tonic modulation mediated by the ambient levels of adenosine present in the extracellular space. Interestingly, this study concluded [80] that the effects of marijuana on hippocampal-dependent memory and cognition in humans might be potentiated during the simultaneous consumption of marijuana and caffeine, which happens frequently. 

By contrast, in the striatum, A_2A_ receptor activation seems to play a permissive role on CB1 receptor mediated depression of excitatory transmission [85]. This effect, however, is related with the activation of postsynaptic A_2A_ receptors, as the effects of cannabinoid agonists were reduced in slices from mice lacking post-synaptic striatal A_2A _receptors.

A_1_ receptor mediated inhibition of excitatory transmission could serve as fine tuning mechanism to decrease the noise of excitatory transmission but also as a break of excessive synaptic activation under pathological conditions and is involved in the regulation of widespread physiological and pathological phenomena, from the regulation of arousal to neuroprotection and seizure susceptibility [81]. 

Activation of A_1_ receptors also inhibits excitatory transmission in the striatum [86], while excitatory A_2A_ receptors participate in NMDA receptor dependent long-term potentiation of synapses between cortical pyramidal neurons and principal striatal medium spiny neurons [87, 88]. Moreover, a recent study showed that co-activation of A_2A_ receptors and fibroblast growth factor (FGF) receptor not only facilitates corticostriatal long term potentiation but also induce morphological plasticity, by inducing increasing spine density and neurite formation by the activation of MAPK/ERK signalling pathway [89].

### Neurochemical Evidence

The first neurochemical evidence for a presynaptic site of action of adenosine was shown on cholinergic terminals in the cerebral cortex and ileal Auerbach plexus [5]. In addition, the study by Vizi and Knoll [5] observed a presynaptic inhibitory effect of nucleotides (ATP, ADP and AMP) via their degradation product, adenosine and discussed the impact of this effect on transmitter release. They described aphenomenon that was different from the generally accepted view that the transmitter of purinergic nerves, ATP, has exclusive actions on P_2_ receptors of the postsynaptic sites [90]. Stimulation of A_2A_ receptors resulted in an increased release of different transmitters, such as glutamate [65, 91] and acetylcholine [92, 93].

Following these pioneering investigations, subsequent studies revealed that the release of almost all transmitters of the CNS is regulated by inhibitory A_1_ and/or facilitatory A_2A_ adenosine receptors (Table **2**). For example, in acute hippocampal slices the electrically evoked release of acetylcholine is subject to dual modulation: it is inhibited by the activation of A_1_ receptors [92, 94] and facilitated by A_2A_ receptors [92, 93]. However, the spatial extension and intensity of the two kind of modulation is not equal. Whilst A_1 _–receptor mediated inhibition is detected in all subregions of the hippocampus, A_2A_ receptor mediated facilitation is manifested only in the CA3 regions and in the dentate gyrus and not the whole hippocampus [92]. It also has been shown that endogenous adenosine, if released as such, preferentially activates A_1 _receptors, whereas it is formed from the released ATP preferentially activates A_2A_ receptors [77]. Moreover aging and other pathological conditions seems to differentially affect inhibitory and facilitatory modulation. The level of endogenous ATP and adenosine is increased during aging [95, 96] and in parallel with these changes the density of A_1_ receptors is decreased [96]. Consequently, a lower degree of inhibitory effect of adenosine agonists on acetylcholine release is detected in hippocampal slices prepared from aged rats [95, 96] but see [97]. On the other hand, the number and functional responsiveness of A_2A_ receptors is increased during aging [95, 97, 98].

In addition to acetylcholine, other transmitters such as glutamate [65, 91] are also subject to dual modulation by A_1_ and A_2A_ receptors, respectively, whereas the release of noradrenaline (NA) [93, 99] and 5-HT [100] appears to be exclusively modulated by A_1_ receptors and that of GABA by A_2A_ receptors [101] in the hippocampus. Adenosine inhibits the release of [^3^H] Glu from hippocampal slice preparation, an effect mediated via A_1_ receptors Fig. (**2**).

Similar rules seems to be valid for the striatum, where the release of acetylcholine is regulated by both inhibitory A_1_ and facilitatory A_2A_ receptors [93, 102] and the release of GABA by A_2A_ receptors [103], while the release of dopamine and 5-HT is modulated by A_1_, but not A_2A_ receptors [93, 104, 105]. 

During ischemia and/or hypoxia, the release of glutamate [106], NA, dopamine (DA) [107] and adenosine [26, 108] was enhanced. During ischemia, the release of transmitter was [Ca^2+^]_o_-independent [11] and mainly originated extrasynaptically from varicose nerve terminals without synaptic contact due to the reverse operation of transporters. Therefore, it seems very unlikely that adenosine is able to reduce transmitter release during ischemia Fig. (**3**). In support of this assumption, we showed that [^3^H]glutamate release from the hippocampus in response to oxygen–deprivation was increased and cannot be inhibited by adenosine and the selective A_1_ receptor antagonist 1,3-dipropyl-8-cyclopentyl-xanthine (DPCPX) [109] failed to enhance the release Fig. (**3**). However, low temperature completely prevented the effect of glucose and oxygen deprivation (OGD) to release glutamate Fig. (**3**), which act nonsynaptically on NR2B receptors Fig. (**4**).

## SYNAPTIC AND NONSYNAPTIC ADENOSINE RECEPTORS

Whilst A_1_ adenosine receptors are highly expressed in many brain regions including the neocortex, hippocampus cerebellum and brain stem, A_2A_ receptors display a more restricted localization, with high expression level in the striatum and olfactory bulb and lower expression in other brain regions [110]. By contrast, the expression of A_2B_ and A_3_ receptors is moderate or low in most areas of the brain [111].

Using quantitative autoradiography, both pre-and postsynaptic localisation of A_1 _receptors was shown in the CA1 region of rat hippocampus [112]. More recent investigations with postembedding immunogold electronmicroscopy revealed that A_1_ receptors are co-localized with P_2Y1_ receptors in hippocampal synapses and distributed to the pre-and postsynaptic membrane as well as to the surrounding astroglial membrane [113]. In glutamatergic synapses of hippocampus, where both A_1_ and A_2A_ receptors are presynaptically expressed [114], the inhibition of synaptic transmission by tonic activation of A_1_ receptors was insurmountable with increasing concentrations of adenosine [79, 115]. There was an interaction between the two adenosine receptors, and activation of presynaptic A_2A _receptors by an agonist may lead to a decrease in the affinity of A_1_ receptors on the terminals [116, 117].

In the basal ganglia, the typical localization of A_2A_ receptors are the dendrites and somata of striatopallidal GABAergic neurons, where it is colocalized with D_2_ receptors. However, immune electronmicroscopy studies showed that it is also expressed, although less abundantly on axons and nerve terminals in both asymmetric and symmetric synapses, which implicate the role of A_2A_ receptors in the modulation of excitatory transmission [118, 119]. Indeed, A_1_ receptors are coexpressed with A_2A_ receptors on the same glutamatergic terminals in the striatum and form heterodimers, when they are co-transfected [120]. Activation of A_2A_ receptors reduce the affinity of the A_1_ receptors for agonists, and provides a switch mechanism by which low and high concentrations of adenosine inhibit and stimulate, respectively, glutamate release [120]. A_1_-A_2A_ heteromers are also recognized as targets for caffeine and it is presumed that chronic caffeine treatment leads to modifications in the function of the A_1_-A_2A_ heteromer that could underlie the strong tolerance to the psychomotor effects of caffeine [121]. On the other hand, the glial expression of A_2A_ receptor is less prevalent than that of A_1_ receptors. 

## CLINICAL ASPECTS

It is generally accepted that adenosine present in extracellular space and acting on A_1 _receptors reduces excitatory transmission. Adenosine has a neuroprotective action in brain injuries, like hypoxia, ischemia, epileptic seizures, and neuroinflammation. Therefore, adenosine A_1_ receptors have been suggested as a potential target for the treatment of neurodegenerative diseases [122]. In agreement with this suggestion it was found that inhibition of A_2A_ receptors improves neuronal recovery on brain injury [123].

### Parkinson’s Disease

Corticolimbic and thalamic glutamatergic neurons and mesencephalic dopaminergic neurons innervate the GABAergic medium-sized spiny neurons of the striatum [124]. These GABAergic pathways are the striatal efferent neurons (“indirect” pathways), which project via the globus pallidus and the subthalamic nucleus to output nuclei (substantia nigra and entopeduncular nucleus). The ‘‘direct’’ pathway sends axons directly to the GABAergic neurons [124]. D_1_-type receptors belong to the ‘‘direct’’ pathway and D_2_-type receptors signal to the ‘‘indirect’’ pathway [124]. The balance between these two pathways is essential for proper functioning of the extrapyramidal motor system [125]. Overactivity of the ‘‘indirect’’ striatal pathway plays an important role in generating parkinsonian symptoms [125]. The degeneration of the nigrostriatal dopaminergic pathway results in striatal dopamine depletion, which consequently impairs the function of the basal ganglia circuits producing akinesia, bradykinesia, tremor, and rigidity [126]. Adenosine exerts its effects in the basal ganglia by acting through A_1_ and A_2A_ adenosine receptors. The A_2A_ receptors are co-localised and interact functionally with D_2_ receptors on the medium-sized spiny neurons of the ‘‘indirect’’ pathway. In addition, A_2A_ receptors also interact with NMDA receptors present on a subpopulation of medium spiny neurons, in a negative way [127, 128].

In the basal ganglia, A_2A_ receptors are prevalently and selectively localised in dendrites, dendritic spines and axons of GABAergic neurons of the indirect pathway projecting from the caudate putamen to the external globus pallidus [118, 119]. Adenosine influences striatal output pathways known to be involved in motor symptoms and the onset of dyskinesia in Parkinson’s disease (PD). Therefore, inhibition of A_2A_ receptors seems to be a potential target for neuroprotection in PD. Indeed, in rodent models of PD, A_2A _antagonism exerts antiparkinsonian actions [119, 129, 130]. Similarly, this treatment proved to be effective against experimentally-induced tremor [131]. The anti-tremor effect might be explained by the fact that A_2A_ receptor antagonists reduced the release of ACh from the striatum [132]. Recently, it was shown that expression level and functionality of A_2A_ adenosine receptors on human lymphocytes correlate with the severity of parkinsonian motor symptoms as scaled by the Unified Parkinson's Disease Rating Scale (UPDRS) [133], implicating that peripheral A_2A_ receptors could also play a role in disease progression. Preclinical studies with istradefylline (an A_2A _selective antagonist) suggest that these antagonists prevent the development of dyskinesia induced by a dopamine agonist [134]. This mechanism offers a rationale for the A_2A_ receptor antagonist treatment as a monotherapy of concurrent administration with levodopa or a dopamine receptor agonist [119, 135]. Unfortunately, clinical trials with istradefylline failed to fulfil expectations [119]. 

Nevertheless, the potential role of A_2A_ receptors in neurodegeneration of the nigro-striatal dopaminergic pathway is supported by a 30-year follow-up study [119, 136], which reported that there was an inverse relationship between consumption of the non-selective adenosine receptor antagonist caffeine and the risk of PD. The neuroprotective action of A_2A_ receptor antagonism is further supported by preclinical studies. Administration of caffeine prevents MPTP-induced damage of dopaminergic terminals [137-139]. DPCPX, an A_1_ receptor antagonist treatment [137] or genetic deletion [137] of A_2A_ receptors proved to be effective against the MPTP model of PD. However, inhibition of A_2A_ receptors expressed on glial cells was also involved in the neuroprotective actions [140]. Cunha *et al.* [141] have shown that A_2A_ receptors in the hippocampus and cerebral cortex are high- affinity receptors, whereas those in the striatum are different. Drugs that were effective in animal models of PD proved to be ineffective as neuroprotective agents in clinical trials [142, 143].

### Ischemia

Glutamate is removed from the extracellular space by nonsynaptically localised glutamate transporters. Astrocytes possess two forms of these transporters, but glutamate is released from glial cells under ischemia due to a reversal of transporter operation. The cascade of events in response to hypoxia or cerebral ischemia is still being debated. However, it is generally accepted that a large amount of glutamate release causes excitotoxic effects Fig. (**4**). The excessive amount of glutamate leads to over-depolarization and the subsequent depression of evoked population potential size, and both A_1_ and A_2A_ receptor activation has been shown to modulate the recovery from the loss of fEPSP in response to glutamatergic insult in the CA1 region of the hippocampus [144]. Evidence has accumulated that neuroprotection is also related to activation of nonsynaptic A_1_ receptors [78]. The A_1_ receptor-mediated effect of adenosine on the release of glutamate has been assumed to play a major protective role against post-ischemic damage [145]. However, this conclusion was drawn from *in vitro *experiments under normoxic conditions e.g. [79, 146]. There is strong neurochemical evidence that the release of transmitters under ischemic conditions is mainly due to the reverse operation of transporters. The release was [Ca^2+^]_o_-independent, did not result from neuronal activity and was not subjected to presynaptic modulation [11]. Lowering the temperature inhibited transporter operation [147, 148] and completely inhibited the extracellular flood of transmitter release, which indicated that the transporter was responsible for the release. To avoid excitotoxicity due to extremely high concentrations of glutamate in the extracellular space and its possible effect on nonsynaptic high affinity NR2B receptors [11], local cooling or inhibition of transporter operation would be an efficient treatment. In addition, it was shown that damage after middle cerebral artery occlusion was not significantly altered by the lack of A_1_ receptor genes [149]. An interesting observation is that A_1_ receptor activation decrease brain energy metabolism [56] and the neuroprotective action of the chemokine fractalkine is mediated by the release of adenosine from microglia and a subsequent action on A_1_ receptors [150, 151]. All these data support the emerging view that neuroprotection, afforded by A_1_ receptor activation, is related with extrasynaptic, rather than synaptic receptors.

### Immune Responses

The original view that the brain is not involved in adaptive and innate immune reactions is no longer accepted. The innate immune system has been implicated in variety of neurodegenerative disorders [152]. Multiple acute and chronic neurodegenerative disorders of the CNS are accompanied by activation of microglia and increased production of proinflammatory cytokines (IL-1, IL-6, IL-12, TNF α) and chemokines [153]. Adenosine possesses anti-inflammatory properties and inhibited proinflammatory cytokine production via activation of A_3_ receptors [154]. However, it has also been shown [155] that A_3_ receptor stimulation had detrimental neurotoxic effects in cerebral ischemia. The case is similar to stimulation of A_2A_ receptors as their activation may produce anti-inflammatory and proinflammatory actions [156]. Interestingly, the balance between the anti- and pro-inflammatory actions of A_2A_ receptor is governed by extracellular glutamate levels in the brain, and the increase in glutamate levels results in a shift of A_2A_ effects from antiinflammatory to proinflammatory direction [157]. Adenosine involvement in immune response is supported by findings that evidence was obtained that there is a significant change in ectonucleotidase activites during experimental autoimmune encephalitis [158].

The combined effects of adenosine on neurotoxicity and inflammatory processes have also led to considerations of its role in Lesch-Nyhan syndrome, and multiple sclerosis [159].

### Epilepsy

It is known that during epileptic seizures, endogenous adenosine accumulates in significant amounts in the extracellular space and suppresses epileptic seizure activity [59, 160, 161]. Adenosine exerts an anticonvulsant effect on the A_1_ adenosine receptors by modulating ionic currents postsynaptically and reducing excitatory neurotransmitter release presynaptically [162]. In addition, A_2A_ receptors modulate the stability of currents mediated by GABA_A_ receptors microtransplanted into Xenopus oocytes from neurosurgically resected epileptic human nervous tissues [163]. In the model of chronic temporal lobe eplilepsy there is an upregulation of the A_1_ receptors [162]. An interesting observation is that a single convulsive episode in early life causes a delayed memory deficit in adulthood accompanied by a glutamatergic synaptotoxicity that was prevented by caffeine or adenosine A_2A_R antagonists [160].

### Psychiatric Disorders

There is growing evidence that A_2A_ receptors able to interact with D_2_ dopamine receptors [164] which are major targets of psychoactive drugs. Therefore these receptors are promising candidate target for therapy applied in mood disorders [165].

## CONCLUDING REMARKS

It is now clear that adenosine is one of the most important neuromodulators in hippocampus and basal ganglia, which regulates a wide variety of neuronal functions pre-, post- and non-synaptically, including synaptic transmission, neuromodulation, glia-neuron interactions and neuroimmunomodulation. Nevertheless, there are a number of aspects which needs further investigation. Despite of the wealth of data on adenosine receptor mediated signaling at the molecular and cellular level, the present knowledge is still limited at the systems level. This holds true to any aspects of purinergic mechanisms including the release and inactivation mechanisms of adenosine and to presynaptic adenosine-receptor mediated responses as well, which are well characterized in *in vitro *systems, but poorly extrapolated to *in vivo* conditions. The progress along this line together with the utilisation of more adequate disease models might lead to the therapeutic utilization of purinergic signaling system, which offer a number of potential target sites for pharmacological intervention in the CNS pathology.

## Figures and Tables

**Fig. (1):**
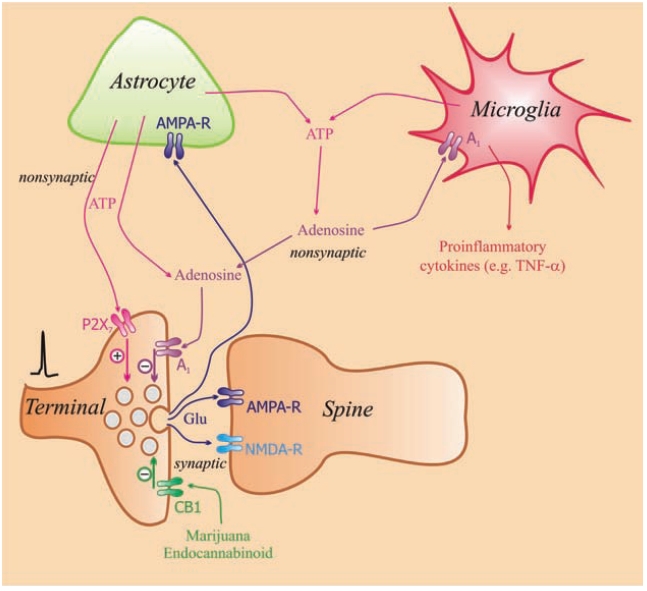
A schematic model of the interaction of P_2X7_, CB1 and A_1_ receptors located on glutamatergic terminals. Activation of P2X_7_ receptors facilitate and those of A_1_ receptors reduces the release of glutamate. Glutamate (Glu) released into synaptic gap activates AMPA and NMDA receptors on the postsynaptic site. ATP released from astrocytes [20] and microglia [172] acts on P_2X7_ receptors located on the terminal of glutamatergic neurons and facilitates the release of Glu ([173] for review see [174]). Adenosine decomposed from ATP acts on A_1_ receptors inhibiting the release of Glu [168, 175]. This inhibitory effect of A_1_ receptor activation may be mediated by inhibiting voltage-dependent Ca^2+^ channels, which reduces Ca transients measured in the bouton [176]. CB1 cannabinoid receptors together with A_1_ receptors are also expressed on glutamatergic terminals [80] and activation of both of these receptors results in a decrease of Glu release. Extremely high concentrations of adenosine act on A_2A_ receptors to increase the release of Glu [77].

**Fig. (2):**
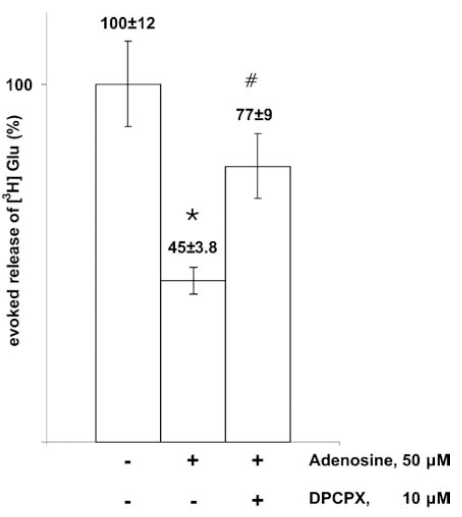
Inhibitory effect of adenosine on glutamate release evoked by axonal stimulation. Hippocampal slice preparation of the rat. For Methods see [147]. Electrical field stimulation was used. Note that A_1_ adenosine receptor antagonist DPCPX prevented the effect of adenosine to reduce Glu release. *, p<0.01 (compared to control); #, p<0.05 (comparison of the effect of adenosine and adenosine plus DPCPX).

**Fig. (3):**
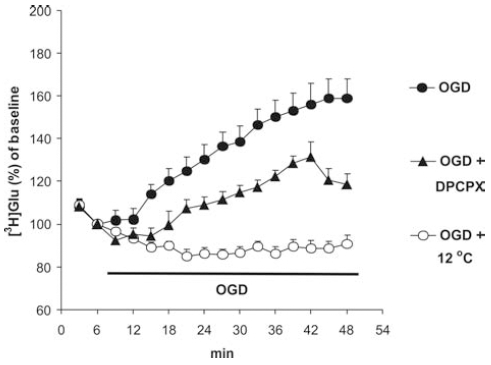
The effect of low temperature (12 °C) and the selective A_1_ receptor antagonist DPCPX (50 nM) on combined oxygen glucose deprivation (OGD)-evoked [^3^H]glutamate efflux from rat hippocampal slices. Hippocampal slices were preloaded with [^3^H]glutamate and then superfused. After a 60-min preperfusion, perfusate samples were collected and the slices were exposed to OGD by the omission of the glucose and the replacement of 95% O_2_+5% CO_2_ with 95% N2 + 5 %CO_2_ from the perfusion solution according to the horizontal bar. Low temperature and DPCPX were applied from 30 min before the beginning of the sample collection period till the end of the sample collection period. The release of glutamate is expressed as a percentage of baseline. The curves show the mean±S.E.M of 16 (OGD), 7 (OGD + 12 °C) and 8 (OGD + DPCPX) experiments.

**Fig. (4):**
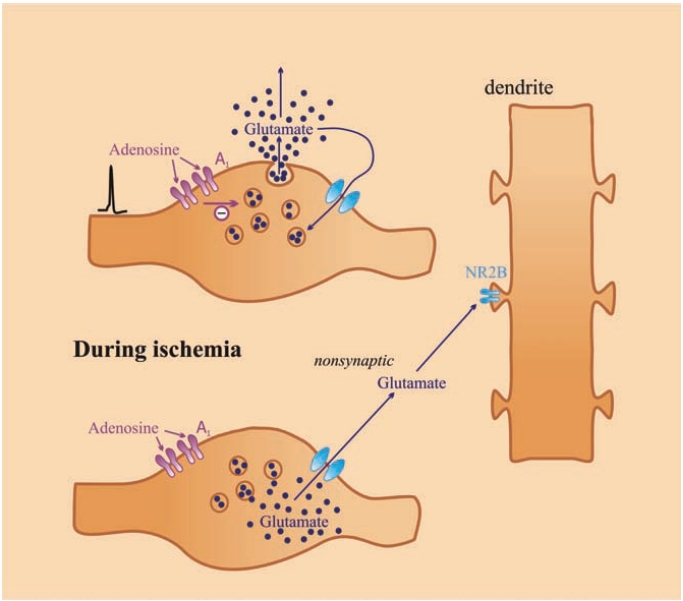
Scheme of exocytosis of glutamate in response to axonal firing and its release during ischemia. Note that adenosine inhibits the release of Glu under physiological condition. This type of release is [Ca^2+^]_o_-dependent. Glutamate is taken up by glutamatergic terminals through plasma membrane transporters. During ischemia the ion gradients that power Glu uptake run down and axonal firing fails to release Glu, but in response to reverse operation of Glu transporter Glu release occurs in [Ca^2+^]_o_–independent way. Glu released in this way diffuses far away and activates non-synaptic NR2B receptors inducing excitotoxicity [11]. Under this condition cooling inhibits the excessive release of Glu.

**Table 1:** Kinetic Parameters of ATP, ADP and AMP Hydrolysis in Rat Hippocampal Slices (Data are Taken From [63])

	K_m_	V_max_
ATP	322 ± 95.24 µM	14.5 ± 3.4 nmol/min/prep
ADP	321.5 ± 94.4 µM	22.6 ± 3.8 nmol/min/prep
AMP	267 ± 52.88 µM	4.99 ± 0.44 nmol/min/prep.

**Table 2:** Neurochemical Evidence of Presynaptic Effect of Adenosine on Transmitter Release in the CNS

	Transmitter Release	Effect	Receptor Mediated	Reference
Cerebral cortex	ACh	inhibition	A_1_	[5, 158]
		facilitation	A_2A_	[166, 167]
Hippocampus	GABA	facilitation	A_2A_	[101]
	ACh	facilitation inhibition	A_2A_ A_1_	[92, 94]
	NA	inhibition	A_1_	[93, 99]
	Glutamate	inhibition	A_1_	[168]
Striatum	Glutamate	inhibition	A_1_	[169]
	5-HT	inhibition	A_1_	[170]
	GABA	inhibition	A_2A_	[103]
	ACh	no effect inhibition	A_2A_ A_1_	[93]
	DA	inhibition no effect	A_1_ A_2A_	[93, 105, 171]

## ABBREVIATIONS

ACh: = Acetylcholine
CNT: = Concentrative transporter
DA: = Dopamine
DPCPX: = 1,3-dipropyl-8-cyclopentylxanthine
E-NPP: = Ecto-nucleotide pyrophosphatase
ENT: = Equilibrative transporter
E-NTPDase: = Ectonucleoside triphosphate diphosphohydrolase
FGF: = Fibroblast growth factor
Glu: = Glutamate
IL-1β: = Interleukin-1β
LPS: = Bacterial lipopolysaccharide
NA: = Noradrenaline
OGD: = Oxygen and glucose deprivation
PD: = Parkinson’s disease
UPDRS: = Unified Parkinson's disease rating scale

## References

[1] Drury A N, Szent-Gyorgyi A (1929). The physiological activity of adenine compounds with especial reference to their action upon the mammalian heart. J. Physiol.

[2] Sattin A, Rall T W (1970). The effect of adenosine and adenine nucleotides on the cyclic adenosine 3', 5'-phosphate content of guinea pig cerebral cortex slices. Mol. Pharmacol.

[3] Pull I, McIlwain H (1972). Adenine derivatives as neurohumoral agents in the brain. The quantities liberated on excitation of superfused cerebral tissues. Biochem. J.

[4] Gustafsson L, Hedqvist P, Fredholm B B, Lundgren G (1978). Inhibition of acetylcholine release in guinea pig ileum by adenosine. Acta Physiol. Scand.

[5] Vizi E S, Knoll J (1976). The inhibitory effect of adenosine and related nucleotides on the release of acetylcholine. Neuroscience.

[6] Burnstock G, Straub R, Bolis L (1978). A Basis for Distinguishing Two Types of Purinergic Receptor. in: *cell Membrane Receptors for Drugs and Hormones: A Multidisciplinary Approach*.

[7] Gu J G, MacDermott A B (1997). Activation of ATP P2X receptors elicits glutamate release from sensory neuron synapses. Nature.

[8] Sanchez-Nogueiro J, Marin-Garcia P, Leon D, Leon-Otegui M, Salas E, Gomez-Villafuertes R, Gualix J, Miras-Portugal M T (2009). Axodendritic fibres. of mouse cerebellar granule neurons exhibit a diversity of functional P2X receptors. Neurochem. Int.

[9] Sperlagh B, Vizi E S (1991). Effect of presynaptic P2 receptor stimulation on transmitter release. J. Neurochem.

[10] Wall M J, Atterbury A, Dale N (2007). Control of basal extracellular adenosine concentration in rat cerebellum. J. Physiol.

[11] Vizi E S, Fekete A, Karoly R, Mike A (2010). Non-synaptic receptors and transporters involved in brain functions and targets of drug treatment. Br. J. Pharmacol.

[12] Bours M J, Swennen E L, Di Virgilio F, Cronstein B N, Dagnelie P C (2006). Adenosine 5'-triphosphate and adenosine as endogenous signaling molecules in immunity and inflammation. Pharmacol. Ther.

[13] Hasko G, Pacher P, Vizi E S, Illes P (2005). Adenosine receptor signaling in the brain immune system. Trends Pharmacol. Sci.

[14] Di Virgilio F (2005). Purinergic mechanism in the immune system: A signal of danger for dendritic cells. Purinergic Signal.

[15] Burnstock G (2007). Purine and pyrimidine receptors. Cell Mol. Life Sci.

[16] Fredholm B B, AP I J, Jacobson K A, Klotz K N, Linden J (2001). International Union of Pharmacology. XXV. Nomenclature and classification of adenosine receptors. Pharmacol. Rev.

[17] Dunwiddie T V, Diao L, Proctor W R (1997). Adenine nucleotides undergo rapid, quantitative conversion to adenosine in the extracellular space in rat hippocampus. J. Neurosci.

[18] Juranyi Z, Sperlagh B, Vizi E S (1999). Involvement of P2 purinoceptors and the nitric oxide pathway in [^3^H]purine outflow evoked by short-term hypoxia and hypoglycemia in rat hippocampal slices. Brain Res.

[19] Sperlágh B (1996). Neuronal synthesis, storage and release of ATP. Semin. Neurosci.

[20] Coco S, Calegari F, Pravettoni E, Pozzi D, Taverna E, Rosa P, Matteoli M, Verderio C (2003). Storage and release of ATP from astrocytes in culture. J. Biol. Chem.

[21] Hamilton N B, Attwell D (2010). Do astrocytes really exocytose neurotransmitters?. Nat. Rev. Neurosci.

[22] Latini S, Pedata F (2001). Adenosine in the central nervous system: release mechanisms and extracellular concentrations. J. Neurochem.

[23] Sperlagh B, Vizi E S, Abbracchio M P, Williams M (2000). Regulation of purine release. In: *Handbook of Experimental Pharmacology*.

[24] Jonzon B, Fredholm B B (1985). Release of purines, noradrenaline, and GABA from rat hippocampal slices by field stimulation. J. Neurochem.

[25] Cunha R A, Vizi E S, Ribeiro J A, Sebastiao A M (1996). Preferential release of ATP and its extracellular catabolism as a source of adenosine upon high- but not low-frequency stimulation of rat hippocampal slices. J. Neurochem.

[26] Pedata F, Latini S, Pugliese A M, Pepeu G (1993). Investigations in to the adenosine outflow from hippocampal slices evoked by ischemia-like conditions. J. Neurochem.

[27] Pedata F, Pazzagli M, Tilli S, Pepeu G (1990). Regional differences in the electrically stimulated release of endogenous and radioactive adenosine and purine derivatives from rat brain slices. Naunyn Schmiedebergs Arch. Pharmacol.

[28] Wieraszko A, Goldsmith G, Seyfried T N (1989). Stimulation-dependent release of adenosine triphosphate from hippocampal slices. Brain Res.

[29] Pedata F, Pazzagli M, Pepeu G (1991). Endogenous adenosine release from hippocampal slices: excitatory amino acid agonists stimulate release, antagonists reduce the electrically-evoked release. Naunyn Schmiedebergs Arch. Pharmacol.

[30] Sperlagh B, Baranyi M, Hasko G, Vizi E S (2004). Potent effect of Interleukin-1 beta to evoke ATP and adenosine release from rat hippocampal slices. J. Neuroimmunol.

[31] Vizi E S, Sperlagh B, Baranyi M (1992). Evidence that ATP released from the postsynaptic site by noradrenaline, is involved in mechanical responses of guinea-pig vas deferens: cascade transmission. Neuroscience.

[32] Ballarin M, Fredholm B B, Ambrosio S,  Mahy N (1991). Extracellular levels of adenosine and its metabolites in the striatum of awake rats: inhibition of uptake and metabolism. Acta Physiol. Scand.

[33] Pazzagli M, Pedata F, Pepeu G (1993). Effect of K+ depolarization, tetrodotoxin, and NMDA receptor inhibition on extracellular adenosine levels in rat striatum. Eur. J. Pharmacol.

[34] Sciotti V M, Park T S, Berne R M, Van Wylen D G (1993). Changes in extracellular adenosine during chemical or electrical brain stimulation. Brain Res.

[35] Van Wylen D G, Park T S, Rubio R, Berne R M (1986). Increases in cerebral Interstitial fluid adenosine concentration during hypoxia, local potassium infusion, and ischemia. J. Cereb. Blood Flow Metab.

[36] Dobolyi A, Reichart A, Szikra T, Szilagyi N, Kekesi A K, Karancsi T, Slegel P, Palkovits M, Juhasz G (1998). Analysis of purine and pyrimidine bases, nucleosides and deoxynucleosides in brain microsamples (microdialysates and micropunches) and cerebrospinal fluid. Neurochem. Int.

[37] Dobolyi A, Szikra T, Kekesi A K, Kovacs Z, Juhasz G (1999). Uridine is released by depolarization and inhibits unit activity in the rat hippocampus. Neuroreport.

[38] Kardos J, Kovacs I, Szarics E, Kovacs R, Skuban N, Nyitrai G, Dobolyi A, Juhasz G (1999). Uridine activates fast transmembrane Ca^2+^ ion fluxes in rat brain homogenates. Neuroreport.

[39] Verderio C, Matteoli M (2001). ATP mediates calcium signaling between astrocytes and microglial cells: modulation by IFN-gamma. J. Immunol.

[40] Wang X, Arcuino G, Takano T, Lin J, Peng W G, Wan P, Li P, Xu Q, Liu Q S, Goldman S A, Nedergaard M (2004). P2X7 receptor inhibition improves recovery after spinal cord injury. Nat. Med.

[41] Wang Y, Roman R, Lidofsky S D, Fitz J G (1996). Autocrine signaling through ATP release represents a novel mechanism for cell volume regulation. Proc. Natl. Acad. Sci. USA.

[42] Hagberg H, Andersson P, Lacarewicz J, Jacobson I, Butcher S, Sandberg M (1987). Extracellular adenosine, inosine, hypoxanthine, and xanthine in relation to tissue nucleotides and purines in rat striatum during transient ischemia. J. Neurochem.

[43] Hisanaga K, Onodera H, Kogure K (1986). Changes in levels of purine and pyrimidine nucleotides during acute hypoxia and recovery in neonatal rat brain. J. Neurochem.

[44] Lutz P L, Kabler S (1997). Release of adenosine and ATP in the brain of the freshwater turtle (Trachemys scripta) during long-term anoxia. Brain Res.

[45] Ferrari D, Chiozzi P, Falzoni S, Hanau S, Di Virgilio F (1997). Purinergic modulation of Interleukin-1 beta release from microglial cells stimulated with bacterial endotoxin. J. Exp. Med.

[46] Sperlagh B, Hasko G, Nemeth Z, Vizi E S (1998). ATP released by LPS increases nitric oxide production in raw 264.7 macrophage cell line via P2Z/P2X7 receptors. Neurochem. Int.

[47] Fowler J C (1993). Changes in extracellular adenosine levels and population spike amplitude during graded hypoxia in the rat hippocampal slice. Naunyn Schmiedebergs Arch. Pharmacol.

[48] Fredholm B B, Dunwiddie T V, Bergman B, Lindstrom K (1984). Levels of adenosine and adenine nucleotides in slices of rat hippocampus. Brain Res.

[49] Frenguelli B G, Llaudet E, Dale N (2003). High-resolution real-time recording with microelectrode biosensors reveals novel aspects of adenosine release during hypoxia in rat hippocampal slices. J. Neurochem.

[50] Frenguelli B G, Wigmore G, Llaudet E, Dale N (2007). Temporal and mechanistic dissociation of ATP and adenosine release during ischaemia in the mammalian hippocampus. J. Neurochem.

[51] Latini S, Corsi C, Pedata F, Pepeu G (1995). The source of brain adenosine outflow during ischemia and electrical stimulation. Neurochem. Int.

[52] Lloyd H G, Fredholm B B (1995). Involvement of adenosine deaminase and adenosine kinase in regulating extracellular adenosine concentration in rat hippocampal slices. Neurochem. Int.

[53] Lloyd H G, Lindstrom K, Fredholm B B (1993). Intracellular formation and release of adenosine from rat hippocampal slices evoked by electrical stimulation or energy depletion. Neurochem. Int.

[54] Andine P, Rudolphi K A, Fredholm B B, Hagberg H (1990). Effect of propentofylline (HWA 285) on extracellular purines and excitatory amino acids in CA1 of rat hippocampus during transient ischaemia. Br. J. Pharmacol.

[55] Dux E, Fastbom J, Ungerstedt U, Rudolphi K, Fredholm B B (1990). Protective effect of adenosine and a novel xanthine derivative propentofylline on the cell damage after bilateral carotid occlusion in the gerbil hippocampus. Brain Res.

[56] Haberg A, Qu H, Haraldseth O, Unsgard G, Sonnewald U (2000). *in vivo* effects of adenosine A1 receptor agonist and antagonist on neuronal and astrocytic Intermediary metabolism studied with ex vivo 13C NMR spectroscopy. J. Neurochem.

[57] Melani A, Pantoni L, Corsi C, Bianchi L, Monopoli A, Bertorelli R, Pepeu G, Pedata F (1999). Striatal outflow of adenosine, excitatory amino acids, gamma-aminobutyric acid, and taurine in awake freely moving rats after middle cerebral artery occlusion: correlations with neurological deficit and histopathological damage. Stroke.

[58] Zetterstrom T, Vernet L, Ungerstedt U, Tossman U, Jonzon B, Fredholm B B (1982). Purine levels in the Intact rat brain. Studies with an implanted perfused hollow fibre. Neurosci. Lett.

[59] Dale N, Frenguelli B G (2009). Release of adenosine and ATP during ischemia and epilepsy. Curr. Neuropharmacol.

[60] Sperlagh B, Szabo G, Erdelyi F, Baranyi M, Vizi E S (2003). Homo- and heteroexchange of adenine nucleotides and nucleosides in rat hippocampal slices by the nucleoside transport system. Br. J. Pharmacol.

[61] Zimmermann H (2006). Ectonucleotidases in the nervous system. Novartis Found. Symp.

[62] Wang T F, Guidotti G (1998). Widespread expression of ecto-apyrase (CD39) in the central nervous system. Brain Res.

[63] Wang T F, Rosenberg P A, Guidotti G (1997). Characterization of brain ecto-apyrase: evidence for only one ecto-apyrase (CD39) gene. Brain Res. Mol. Brain Res.

[64] Sperlagh B, Vizi E S (2007). Extracellular Interconversion of nucleotides reveals an ecto-adenylate kinase activity in the rat hippocampus. Neurochem. Res.

[65] Cunha R A (2001). Adenosine as a neuromodulator and as a homeostatic regulator in the nervous system: different roles, different sources and different receptors. Neurochem. Int.

[66] Grondal E J, Janetzko A, Zimmermann H (1988). Monospecific antiserum against 5'-nucleotidase from Torpedo electric organ: immunocytochemical distribution of the enzyme and its association with Schwann cell membranes. Neuroscience.

[67] Schoen S W, Graeber M B, Reddington M, Kreutzberg G W (1987). Light and electron microscopical immunocytochemistry of 5'-nucleotidase in rat cerebellum. Histochemistry.

[68] Cunha R A, Sebastiao A M, Ribeiro J A (1992). Ecto-5'-nucleotidase is associated with cholinergic nerve terminals in the hippocampus but not in the cerebral cortex of the rat. J. Neurochem.

[69] James S, Richardson P J (1993). Production of adenosine from extracellular ATP at the striatal cholinergic synapse. J. Neurochem.

[70] Sperlagh B, Vizi E S, Hamon M (2008). ATP Mediated Signaling in the Nervous System. Handbook of Neurochemistry and Molecular Biology, Neurotransmitter systems.

[71] Dare E, Schulte G, Karovic O, Hammarberg C, Fredholm B B (2007). Modulation of glial cell functions by adenosine receptors. Physiol. Behav.

[72] Cass C E, Young J D, Baldwin S A, Cabrita M A, Graham K A, Griffiths M, Jennings L L, Mackey J R, Ng A M, Ritzel M W, Vickers M F, Yao S Y (1999). Nucleoside transporters of mammalian cells. Pharm. Biotechnol.

[73] Thorn J A, Jarvis S M (1996). Adenosine transporters. Gen. Pharmacol.

[74] Pascual O, Casper K B, Kubera C, Zhang J, Revilla-Sanchez R, Sul J Y, Takano H, Moss S J, McCarthy K, Haydon P G (2005). Astrocytic purinergic signaling coordinates synaptic networks. Science.

[75] Latini S, Bordoni F, Pedata F, Corradetti R (1999). Extracellular adenosine concentrations during *in vitro* ischaemia in rat hippocampal slices. Br. J. Pharmacol.

[76] Sebastiao A M, Ribeiro J A (2009). Tuning and fine-tuning of synapses with adenosine. Curr. Neuropharmacol.

[77] Cunha R A, Correia-de-Sa P, Sebastiao A M, Ribeiro J A (1996). Preferential activation of excitatory adenosine receptors at rat hippocampal and neuromuscular synapses by adenosine formed from released adenine nucleotides. Br. J. Pharmacol.

[78] Cunha R A (2008). Different cellular sources and different roles of adenosine: A1 receptor-mediated inhibition through astrocytic-driven volume transmission and synapse-restricted A2A receptor-mediated facilitation of plasticity. Neurochem. Int.

[79] Sebastiao A M, Stone T W, Ribeiro J A (1990). The inhibitory adenosine receptor at the neuromuscular junction and hippocampus of the rat: antagonism by 1,3,8-substituted xanthines. Br. J. Pharmacol.

[80] Hoffman A F, Laaris N, Kawamura M, Masino S A, Lupica C R (2010). Control of cannabinoid CB1 receptor function on glutamate axon terminals by endogenous adenosine acting at A1 receptors. J. Neurosci.

[81] Dunwiddie T V, Masino S A (2001). The role and regulation of adenosine in the central nervous system. Annu. Rev. Neurosci.

[82] Straiker A J, Borden C R, Sullivan J M (2002). G-protein alpha subunit isoforms couple differentially to receptors that mediate presynaptic inhibition at rat hippocampal synapses. J. Neurosci.

[83] Serrano A, Haddjeri N, Lacaille J C, Robitaille R (2006). GABAergic network activation of glial cells underlies hippocampal heterosynaptic depression. J. Neurosci.

[84] Zhang J M, Wang H K, Ye C Q, Ge W, Chen Y, Jiang Z L, Wu C P, Poo M M, Duan S (2003). ATP released by astrocytes mediates glutamatergic activity-dependent heterosynaptic suppression. Neuron.

[85] Tebano M T, Martire A, Chiodi V, Pepponi R, Ferrante A, Domenici M R, Frank C, Chen J F, Ledent C, Popoli P (2009). Adenosine A2A receptors enable the synaptic effects of cannabinoid CB1 receptors in the rodent striatum. J. Neurochem.

[86] Malenka R C, Kocsis J D (1988). Presynaptic actions of carbachol and adenosine on corticostriatal synaptic transmission studied *in vitro*. J. Neurosci.

[87] Lovinger D M (2010). Neurotransmitter roles in synaptic modulation, plasticity and learning in the dorsal striatum. Neuropharmacology.

[88] Shen W, Flajolet M, Greengard P, Surmeier D J (2008). Dichotomous dopaminergic control of striatal synaptic plasticity. Science.

[89] Flajolet M, Wang Z, Futter M, Shen W, Nuangchamnong N, Bendor J, Wallach I, Nairn A C, Surmeier D J, Greengard P (2008). FGF acts as a co-transmitter through adenosine A(2A) receptor to regulate synaptic plasticity. Nat. Neurosci.

[90] Burnstock G (1972). Purinergic nerves. Pharmacol. Rev.

[91] Fredholm B B, Chen J F, Cunha R A, Svenningsson P, Vaugeois J M (2005). Adenosine and brain function. Int. Rev. NeuroBiol.

[92] Cunha R A, Milusheva E, Vizi E S, Ribeiro J A, Sebastiao A M (1994). Excitatory and inhibitory effects of A1 and A2A adenosine receptor activation on the electrically evoked [^3^H]acetylcholine release from different areas of the rat hippocampus. J. Neurochem.

[93] Jin S, Fredholm B B (1997). Adenosine A2A receptor stimulation increases release of acetylcholine from rat hippocampus but not striatum, and does not affect catecholamine release. Naunyn Schmiedebergs Arch. Pharmacol.

[94] Jackisch R, Strittmatter H, Kasakov L, Hertting G (1984). Endogenous adenosine as a modulator of hippocampal acetylcholine release. Naunyn Schmiedebergs Arch. Pharmacol.

[95] Cunha R A, Almeida T, Ribeiro J A (2001). Parallel modification of adenosine extracellular metabolism and modulatory action in the hippocampus of aged rats. J. Neurochem.

[96] Sperlagh B, Zsilla G, Baranyi M, Kekes-Szabo A, Vizi E S (1997). Age-dependent changes of presynaptic neuromodulation via A1-adenosine receptors in rat hippocampal slices. Int. J. Dev. Neurosci.

[97] Rodrigues R J, Canas P M, Lopes L V, Oliveira C R, Cunha R A (2008). Modification of adenosine modulation of acetylcholine release in the hippocampus of aged rats. NeuroBiol. Aging.

[98] Lopes L V, Cunha R A, Ribeiro J A (1999). Increase in the number, G protein coupling, and efficiency of facilitatory adenosine A2A receptors in the limbic cortex, but not striatum, of aged rats. J. Neurochem.

[99] Allgaier C, Greber R, Hertting G (1991). Studies on the Interaction between presynaptic alpha 2-adrenoceptors and adenosine A1 receptors located on noradrenergic nerve terminals. Naunyn Schmiedebergs Arch. Pharmacol.

[100] Okada M, Kawata Y, Kiryu K, Mizuno K, Wada K, Tasaki H, Kaneko S (1997). Effects of adenosine receptor subtypes on hippocampal extracellular serotonin level and serotonin reuptake activity. J. Neurochem.

[101] Cunha R A, Ribeiro J A (2000). Purinergic modulation of [(3)H]GABA release from rat hippocampal nerve terminals. Neuropharmacology.

[102] Kirkpatrick K A, Richardson P J (1993). Adenosine receptor-mediated modulation of acetylcholine release from rat striatal synaptosomes. Br. J. Pharmacol.

[103] Kirk I P, Richardson P J (1995). Inhibition of striatal GABA release by the adenosine A2a receptor is not mediated by increases in cyclic AMP. J. Neurochem.

[104] Okada M, Mizuno K, Kaneko S (1996). Adenosine A1 and A2 receptors modulate extracellular dopamine levels in rat striatum. Neurosci. Lett.

[105] Zetterstrom T, Fillenz M (1990). Adenosine agonists can both inhibit and enhance *in vivo* striatal dopamine release. Eur. J. Pharmacol.

[106] Nicholls D, Attwell D (1990). The release and uptake of excitatory amino acids. Trends Pharmacol. Sci.

[107] Milusheva E, Doda M, Pasztor E, Lajtha A, Sershen H, Vizi E S (1992). Regulatory Interactions among axon terminals affecting the release of different transmitters from rat striatal slices under hypoxic and hypoglycemic conditions. J. Neurochem.

[108] Dale N, Pearson T, Frenguelli B G (2000). Direct measurement of adenosine release during hypoxia in the CA1 region of the rat hippocampal slice. J. Physiol.

[109] Sperlagh B, Zsilla G, Baranyi M, Illes P, Vizi E S (2007). Purinergic modulation of glutamate release under ischemic-like conditions in the hippocampus. Neuroscience.

[110] Sebastiao A M, Ribeiro J A (2009). Adenosine receptors and the central nervous system. Handb Exp Pharmacol.

[111] Dixon A K, Gubitz A K, Sirinathsinghji D J, Richardson P J, Freeman T C (1996). Tissue distribution of adenosine receptor mRNAs in the rat. Br. J. Pharmacol.

[112] Deckert J, Jorgensen M B (1988). Evidence for pre- and postsynaptic localization of adenosine A1 receptors in the CA1 region of rat hippocampus: a quantitative autoradiographic study. Brain Res.

[113] Tonazzini I, Trincavelli M L, Storm-Mathisen J, Martini C, Bergersen L H (2007). Co-localization and functional cross-talk between A1 and P2Y1 purine receptors in rat hippocampus. Eur. J. Neurosci.

[114] Rebola N, Canas P M, Oliveira C R, Cunha R A (2005). Different synaptic and subsynaptic localization of adenosine A2A receptors in the hippocampus and striatum of the rat. Neuroscience.

[115] Johansson B, Halldner L, Dunwiddie T V, Masino S A, Poelchen W, Gimenez-Llort L, Escorihuela R M, Fernandez-Teruel A, Wiesenfeld-Hallin Z, Xu X J, Hardemark A, Betsholtz C, Herlenius E, Fredholm B B (2001). Hyperalgesia, anxiety, and decreased hypoxic neuroprotection in mice lacking the adenosine A1 receptor. Proc. Natl. Acad. Sci. USA.

[116] Dixon A K, Widdowson L, Richardson P J (1997). Desensitisation of the adenosine A1 receptor by the A2A receptor in the rat striatum. J. Neurochem.

[117] O'Kane E M, Stone T W (1998). Interaction between adenosine A1 and A2 receptor-mediated responses in the rat hippocampus *in vitro*. Eur. J. Pharmacol.

[118] Hettinger B D, Lee A, Linden J, Rosin D L (2001). Ultrastructural localization of adenosine A2A receptors suggests multiple cellular sites for modulation of GABAergic neurons in rat striatum. J. Comp. Neurol.

[119] Jenner P, Mori A, Hauser R, Morelli M, Fredholm B B, Chen J F (2009). Adenosine, adenosine A 2A antagonists, and Parkinson's disease. Parkinsonism Relat. Disord.

[120] Ciruela F, Casado V, Rodrigues R J, Lujan R, Burgueno J, Canals M, Borycz J, Rebola N, Goldberg S R, Mallol J, Cortes A, Canela E I, Lopez-Gimenez J F, Milligan G, Lluis C, Cunha R A, Ferre S, Franco R (2006). Presynaptic control of striatal glutamatergic neurotransmission by adenosine A1-A2A receptor heteromers. J. Neurosci.

[121] Ferre S, Ciruela F, Borycz J, Solinas M, Quarta D, Antoniou K, Quiroz C, Justinova Z, Lluis C, Franco R, Goldberg S R (2008). Adenosine A1-A2A receptor heteromers: new targets for caffeine in the brain. Front. Biosci.

[122] Ribeiro J A (2003). Adenosine receptors in the nervous system: pathophysiological implications. Prog. NeuroBiol.

[123] Cunha R A (2005). Neuroprotection by adenosine in the brain: From A(1) receptor activation to A (2A) receptor blockade. Purinergic Signal.

[124] Gerfen C R, Paxinos G (2004). Basal ganglia. The Rat Nervous System. Elsevier Academic: Amsterdam.

[125] Graybiel A M (2000). The basal ganglia. Curr. Biol.

[126] Singh N, Pillay V, Choonara Y E (2007). Advances in the treatment of Parkinson's disease. Prog. Neurobiol.

[127] Gerevich Z, Wirkner K, Illes P (2002). Adenosine A2A receptors inhibit the N-methyl-D-aspartate component of excitatory synaptic currents in rat striatal neurons. Eur. J. Pharmacol.

[128] Norenberg W, Wirkner K, Illes P (1997). Effect of adenosine and some of its structural analogues on the conductance of NMDA receptor channels in a subset of rat neostriatal neurones. Br. J. Pharmacol.

[129] Hauber W, Neuscheler P, Nagel J, Muller C E (2001). Catalepsy induced by a blockade of dopamine D1 or D2 receptors was reversed by a concomitant blockade of adenosine A(2A) receptors in the caudate-putamen of rats. Eur. J. Neurosci.

[130] Shiozaki S, Ichikawa S, Nakamura J, Kitamura S, Yamada K, Kuwana Y (1999). Actions of adenosine A2A receptor antagonist KW-6002 on drug-induced catalepsy and hypokinesia caused by reserpine or MPTP. Psychopharmacology, (Berl).

[131] Correa M, Wisniecki A, Betz A, Dobson D R, O'Neill M F, O'Neill M J, Salamone J D (2004). The adenosine A2A antagonist KF17837 reverses the locomotor suppression and tremulous jaw movements induced by haloperidol in rats: possible relevance to parkinsonism. Behav. Brain Res.

[132] Kurokawa M, Koga K, Kase H, Nakamura J, Kuwana Y (1996). Adenosine A2a receptor-mediated modulation of striatal acetylcholine release *in vivo*. J. Neurochem.

[133] Varani K, Vincenzi F, Tosi A, Gessi S, Casetta I, Granieri G, Fazio P, Leung E, MacLennan S, Granieri E, Borea P A (2009). A2A adenosine receptor overexpression and functionality, as well as TNF-alpha levels, correlate with motor symptoms in Parkinson's disease. FASEB J.

[134] Bibbiani F, Oh J D, Petzer J P, Castagnoli N , Chen J F, Schwarzschild M A, Chase T N (2003). A2A antagonist prevents dopamine agonist-induced motor complications in animal models of Parkinson's disease. Exp. Neurol.

[135] Morelli M, Di Paolo T, Wardas J, Calon F, Xiao D, Schwarzschild M A (2007). Role of adenosine A2A receptors in parkinsonian motor impairment and l-DOPA-induced motor complications. Prog. Neurobiol.

[136] Ross G W, Abbott R D, Petrovitch H, Morens D M, Grandinetti A, Tung K H, Tanner C M, Masaki K H, Blanchette P L, Curb J D, Popper J S, White L R (2000). Association of coffee and caffeine Intake with the risk of Parkinson disease. JAMA.

[137] Chen J F, Moratalla R, Impagnatiello F, Grandy D K, Cuellar B, Rubinstein M, Beilstein M A, Hackett E, Fink J S, Low M J, Ongini E, Schwarzschild M A (2001). The role of the D(2) dopamine receptor (D(2)R) in A(2A) adenosine receptor (A(2A)R)-mediated behavioral and cellular responses as revealed by A(2A) and D(2) receptor knockout mice. Proc. Natl. Acad. Sci. USA.

[138] Ikeda K, Kurokawa M, Aoyama S, Kuwana Y (2002). Neuroprotection by adenosine A2A receptor blockade in experimental models of Parkinson's disease. J. Neurochem.

[139] Xu K, Xu Y H, Chen J F, Schwarzschild M A (2002). Caffeine's neuroprotection against 1-methyl-4-phenyl-1,2,3,6-tetrahydro-pyridine toxicity shows no tolerance to chronic caffeine administration in mice. Neurosci. Lett.

[140] Yu L, Shen H Y, Coelho J E, Araujo I M, Huang Q Y, Day Y J, Rebola N, Canas P M, Rapp E K, Ferrara J, Taylor D, Muller C E, Linden J, Cunha R A, Chen J F (2008). Adenosine A2A receptor antagonists exert motor and neuroprotective effects by distinct cellular mechanisms. Ann. Neurol.

[141] Cunha R A, Johansson B, Constantino M D, Sebastiao A M, Fredholm B B (1996). Evidence for high-affinity binding sites for the adenosine A2A receptor agonist [^3^H] CGS 21680 in the rat hippocampus and cerebral cortex that are different from striatal A2A receptors. Naunyn Schmiedebergs Arch. Pharmacol.

[142] Botsakis K, Pavlou O, Poulou P D, Matsokis N, Angelatou F (2009). Blockade of adenosine A2A receptors downregulates DARPP-32 but increases ERK1/2 activity in striatum of dopamine deficient "weaver" mouse. Neurochem. Int.

[143] Morelli M, Carta A R, Jenner P (2009). Adenosine A2A receptors and Parkinson's disease. Handb. Exp. Pharmacol.

[144] Ferguson A L, Stone T W (2010). Glutamate-induced depression of EPSP-spike coupling in rat hippocampal CA1 neurons and modulation by adenosine receptors. Eur. J. Neurosci.

[145] Nobre H V, Cunha G M, de Vasconcelos L M, Magalhaes H I, Oliveira Neto R N, Maia F D, de Moraes M O, Leal L K, Viana G S (2009). Caffeine and CSC, adenosine A2A antagonists, offer neuroprotection against 6-OHDA-induced neurotoxicity in rat mesencephalic cells. Neurochem. Int.

[146] Arrigoni E, Crocker A J, Saper C B, Greene R W, Scammell T E (2005). Deletion of presynaptic adenosine A1 receptors impairs the recovery of synaptic transmission after hypoxia. Neuroscience.

[147] Vizi E S (1998). Different temperature dependence of carrier-mediated (cytoplasmic) and stimulus-evoked (exocytotic) release of transmitter: a simple method to separate the two types of release. Neurochem. Int.

[148] Vizi E S, Sperlagh B (1999). Separation of carrier mediated and vesicular release of GABA from rat brain slices. Neurochem. Int.

[149] Olsson T, Cronberg T, Rytter A, Asztely F, Fredholm B B, Smith M L, Wieloch T (2004). Deletion of the adenosine A1 receptor gene does not alter neuronal damage following ischaemia *in vivo* or *in vitro*. Eur. J. Neurosci.

[150] Lauro C, Di Angelantonio S, Cipriani R, Sobrero F, Antonilli L, Brusadin V, Ragozzino D, Limatola C (2008). Activity of adenosine receptors type 1 Is required for CX3CL1-mediated neuroprotection and neuromodulation in hippocampal neurons. J. Immunol.

[151] Lauro C, Cipriani R, Catalano M, Trettel F, Chece G, Brusadin V, Antonilli L, van Rooijen N, Eusebi F, Fredholm B B, Limatola C (2010). Adenosine A1 receptors and microglial cells mediate CX3CL1-induced protection of hippocampal neurons against Glu-induced death. Neuropsychopharmacology.

[152] Szelenyi J (2001). Cytokines and the central nervous system. Brain Res. Bull.

[153] Geiger J D, Buscemi L, Fotheringham JA, Bruce N, Gyӧrgy  Haskό, Bruce  N (2007). Role of Adenosine in the Control of Inflammatory Events Associated with Acute and Chronic Neurodegenerative Disorders. Adenosine receptors therapeutic aspects for inflammatory and immune disorders.

[154] Hasko G, Nemeth Z H, Vizi E S, Salzman A L, Szabo C (1998). An agonist of adenosine A3 receptors decreases Interleukin-12 and Interferon-gamma production and prevents lethality in endotoxemic mice. Eur. J. Pharmacol.

[155] Wei J, Li H, Qu W, Gao Q (2009). Molecular docking study of A(3) adenosine receptor antagonists and pharmacophore-based drug design. Neurochem. Int.

[156] Pedata F, Corsi C, Melani A, Bordoni F, Latini S (2001). Adenosine extracellular brain concentrations and role of A2A receptors in ischemia. Ann. N. Y. Acad. Sci.

[157] Dai S S, Zhou Y G, Li W, An J H, Li P, Yang N, Chen X Y, Xiong R P, Liu P, Zhao Y, Shen H Y, Zhu P F, Chen J F (2010). Local glutamate level dictates adenosine A2A receptor regulation of neuroinflammation and traumatic brain injury. J. Neurosci.

[158] Broad R M (1995). A1, but not A2A, Adenosine receptors modulate electrically stimulated [14C]acetylcholine release from rat cortex. J. Pharm. Exp. Therap.

[159] Stone T W, Ceruti S, Abbracchio M P (2009). Adenosine receptors and neurological disease: neuroprotection and neurodegeneration. Handb. Exp. Pharmacol.

[160] Cognato G P, Agostinho P M, Hockemeyer J, Muller C E, Souza D O, Cunha R A (2009). Caffeine and an adenosine A(2A) receptor antagonist prevent memory impairment and synaptotoxicity in adult rats triggered by a convulsive episode in early life. J. Neurochem.

[161] During M J, Spencer D D (1992). Adenosine: a potential mediator of seizure arrest and postictal refractoriness. Ann. Neurol.

[162] Ohta Y, Nariai T, Kurumaji A, Hirakawa K, Ohno K (2010). Increased binding of inhibitory neuronal receptors in the hippocampus in kainate-treated rats with spontaneous limbic seizures. J. Clin. Neurosci.

[163] Roseti C, Martinello K, Fucile S, Piccari V, Mascia A, Di Gennaro G, Quarato P P, Manfredi M, Esposito V, Cantore G, Arcella A, Simonato M, Fredholm B B, Limatola C, Miledi R, Eusebi F (2008). Adenosine receptor antagonists alter the stability of human epileptic GABAA receptors. Proc. Natl. Acad. Sci. USA.

[164] Ferre S, Fredholm B B, Morelli M, Popoli P, Fuxe K (1997). Adenosine-dopamine receptor-receptor Interactions as an Integrative mechanism in the basal ganglia. Trends Neurosci.

[165] Cunha R A, Ferre S, Vaugeois J M, Chen J F (2008). Potential therapeutic Int. erest of adenosine A2A receptors in psychiatric disorders. Curr. Pharm. Des.

[166] Spignoli G, Pedata F, Pepeu G (1984). A1 and A2 adenosine receptors modulate acetylcholine release from brain slices. Eur. J. Pharmacol.

[167] Marcoli M, Raiteri L, Bonfanti A, Monopoli A, Ongini E, Raiteri M, Maura G (2003). Sensitivity to selective adenosine A1 and A2A receptor antagonists of the release of glutamate induced by ischemia in rat cerebrocortical slices. Neuropharmacology.

[168] Fastbom J, Fredholm B B (1985). Inhibition of [^3^H] glutamate release from rat hippocampal slices by L-phenylisopropyladenosine. Acta Physiol. Scand.

[169] Ambrosio A F, Malva J O, Carvalho A P, Carvalho C M (1997). Inhibition of N-,P/Q- and other types of Ca^2+^ channels in rat hippocampal nerve terminals by the adenosine A1 receptor. Eur. J. Pharmacol.

[170] Feuerstein T J, Hertting G, Jackisch R (1985). Modulation of hippocampal serotonin (5-HT) release by endogenous adenosine. Eur. J. Pharmacol.

[171] Michaelis M L, Michaelis E K, Myers S L (1979). Adenosine modulation of synaptosomal dopamine release. Life Sci.

[172] Honda S, Sasaki Y, Ohsawa K, Imai Y, Nakamura Y, Inoue K, Kohsaka S (2001). Extracellular ATP or ADP induce chemotaxis of cultured microglia through Gi/o-coupled P2Y receptors. J. Neurosci.

[173] Bennett G C, Boarder M R (2000). The effect of nucleotides and adenosine on stimulus-evoked glutamate release from rat brain cortical slices. Br. J. Pharmacol.

[174] Sperlagh B, Vizi E S, Wirkner K, Illes P (2006). P2X7 receptors in the nervous system. Prog. NeuroBiol.

[175] Zhang C, Schmidt J T (1998). Adenosine A1 receptors mediate retinotectal presynaptic inhibition: uncoupling by C-kinase and role in LTP during regeneration. J. NeuroPhysiol.

[176] Zhang W, Linden D J (2009). Neuromodulation at single presynaptic boutons of cerebellar parallel fibers is determined by bouton size and basal action potential-evoked Ca transient amplitude. J. Neurosci.

